# Pain in women: relevance of history of iron deficiency: comment on Laughey et al. (2025)

**DOI:** 10.1097/PR9.0000000000001383

**Published:** 2025-12-23

**Authors:** G. David Champion, Tiina Jaaniste, Aidan Christopher Tan

**Affiliations:** a Department of Pain Medicine, Sydney Children's Hospital, Randwick, NSW, Australia; b School of Clinical Medicine, Paediatrics and Child Health, University of New South Wales, Kensington, NSW, Australia

## Letter to the Editor:

Laughey et al.^2^ reviewed the biological, psychological, and sociological influences on pain differences between women and men. Their explanations for sex and gender differences in pain sensitivity, central sensitization, transition to chronic pain, and prevalence of pain conditions and explanations offered by Smith et al.^5^ and Liu et al.,^3^ have not included the potential influence of a history of iron deficiency. Iron deficiency disproportionately affects adolescent and adult females and is associated, probably causally, with their greater pain sensitivity and vulnerability to multiple primary and chronic pain conditions in paediatric and adult samples.^1^ This study on iron deficiency and pain was not published at the time of the Laughey et al. review.

In a sample^1^ of 1,817 adult twin family members who responded to questions about a history of iron deficiency (doctor-confirmed, duration >3 months), 11 (4.3%) of the 259 children (aged 3–11) reported a history of iron deficiency, with no significant difference between sexes. Of the 232 adolescents (12–17 years), 22 (9.5%) reported a history of iron deficiency, comprising 17.0% of females and 3.8% of males (*P* = 0.0007). There were 1,326 adult (>17 years) responders with a history of iron deficiency, of whom 38.7% were female and 2.4% male (*P* < 0.0001). This age and gender distribution was comparable with an Australian population survey of a history of iron deficiency.^4^ The disparity between females and males emerged during adolescence and was substantial in adults. Virtually all responders with iron deficiency had taken iron supplements. Thus, sampling iron biomarkers including serum ferritin would not have revealed the pain associations summarized below.

A history of iron deficiency as predictor was statistically significantly associated with a lifetime history (at least 3 months) of migraine, nonmigraine headaches, recurrent abdominal pain, dysmenorrhoea, diverse chronic pain conditions, and with the number of pain conditions.^1^ These results were consistent with our cited 2 pediatric studies and with our study of dysmenorrhea in adolescents and young women, which additionally showed that iron deficiency was associated with sensitivity to pain. A history of iron deficiency was associated with anxiety and depression,^1^ and, in our recently reported research, with multisensory sensitivity. We cited 14 publications^1^ showing associations between iron deficiency and individual pain conditions. The history of iron deficiency affected pain sensitivity and vulnerability probably mainly through its known influences on anxiety, depression, impaired sleep, and as we have recently reported, multisensory sensitivity. Two cited mouse experiments raised the probability of a direct effect of iron deficiency on the central nervous system.^1^

In conclusion, there is much higher prevalence of iron deficiency in female adolescents and adults, and there is increasing evidence that a history of or current iron deficiency is associated with pain sensitivity and vulnerability. It is reasonable to hypothesize that a history of iron deficiency, even after iron supplementation, contributes to the propensity to higher pain sensitivity, central sensitization, and vulnerability to multiple pain conditions in females than in males. Assuming there will be further evidence for our concepts, prevention of iron deficiency, especially in females, is a high priority.

## Disclosures

The authors have no conflicts of interest to declare. The authors received no external funding for this work.
